# mPies: a novel metaproteomics tool for the creation of relevant protein databases and automatized protein annotation

**DOI:** 10.1186/s13062-019-0253-x

**Published:** 2019-11-14

**Authors:** Johannes Werner, Augustin Géron, Jules Kerssemakers, Sabine Matallana-Surget

**Affiliations:** 1 Department of Biological Oceanography, Leibniz Institute of Baltic Sea Research, Seestraße 15, D-18119 Rostock, Germany; 2 Division of Biological and Environmental Sciences, Faculty of Natural Sciences, University of Stirling, Stirling, FK9 4LA UK; 3 Proteomic and Microbiology Department, University of Mons, 7000 Mons, Belgium; 4 Omics IT and Data Management, German Cancer Research Center, 69120 Heidelberg, Germany

**Keywords:** Bioinformatics, Metaproteomics, Microbial ecology, Protein annotation, Protein search database

## Abstract

**Abstract:**

Metaproteomics allows to decipher the structure and functionality of microbial communities. Despite its rapid development, crucial steps such as the creation of standardized protein search databases and reliable protein annotation remain challenging. To overcome those critical steps, we developed a new program named mPies (**m**eta**P**roteomics **i**n **e**nvironmental **s**ciences). mPies allows the creation of protein databases derived from assembled or unassembled metagenomes, and/or public repositories based on taxon IDs, gene or protein names. For the first time, mPies facilitates the automatization of reliable taxonomic and functional consensus annotations at the protein group level, minimizing the well-known protein inference issue, which is commonly encountered in metaproteomics. mPies’ workflow is highly customizable with regards to input data, workflow steps, and parameter adjustment. mPies is implemented in Python 3/Snakemake and freely available on GitHub: https://github.com/johanneswerner/mPies/.

**Reviewer:**

This article was reviewed by Dr. Wilson Wen Bin Goh.

## Implementation

### Background

Metaproteomics is a valuable method to link the taxonomic diversity and functions of microbial communities [1]. However, the use of metaproteomics still faces methodological challenges and lacks of standardisation [2]. The creation of relevant protein search databases and protein annotation remain hampered by the inherent complexity of microbial communities [3].

Protein search databases can be created based on reads or contigs derived from metagenomic and/or metatranscriptomic data [4, 5]. Public repositories such as Ensembl [6], NCBI [7] or UniProtKB [8] can also be used as search databases but it is necessary to apply relevant filters (e.g. based on the habitat or the taxonomic composition) in order to decrease computation time and false discovery rate [4]. Until now, no tool exists that either creates taxonomic or functional subsets of public repositories or combines different protein databases in order to optimize the total number of identified proteins.

The so-called *protein inference issue* occurs when the same peptide sequence is found in multiple proteins, thus leading to inaccurate taxonomic and functional interpretation [9]. To address this issue, protein identification software tools such as ProteinPilot (Pro Group algorithm) [10], Prophane [11] or MetaProteomeAnalyzer [12] perform automatic grouping of homologous protein sequences. Interpreting protein groups can be challenging especially in complex microbial community where redundant proteins can be found in a broad taxonomic range. A well-known strategy to deal with homologous protein sequences is to calculate the lowest common ancestor (LCA). For instance, MEGAN performs taxonomic binning by assigning sequences on the nodes of the NCBI taxonomy and calculates the LCA on the best alignment hit [13]. However, another crucial challenge related to protein annotation still remains: protein sequences annotation often relies on alignment programs automatically retrieving the first hit only [14]. The reliability of this approach is hampered by the existence of taxonomic and functional discrepancies among the top alignment results with very low e-values [5]. Here, we present mPies, a new highly customizable program that allows the creation of protein search databases and performs post-search protein consensus annotation, thus facilitating biological interpretation.

### Workflow design

**Fig. 1:**
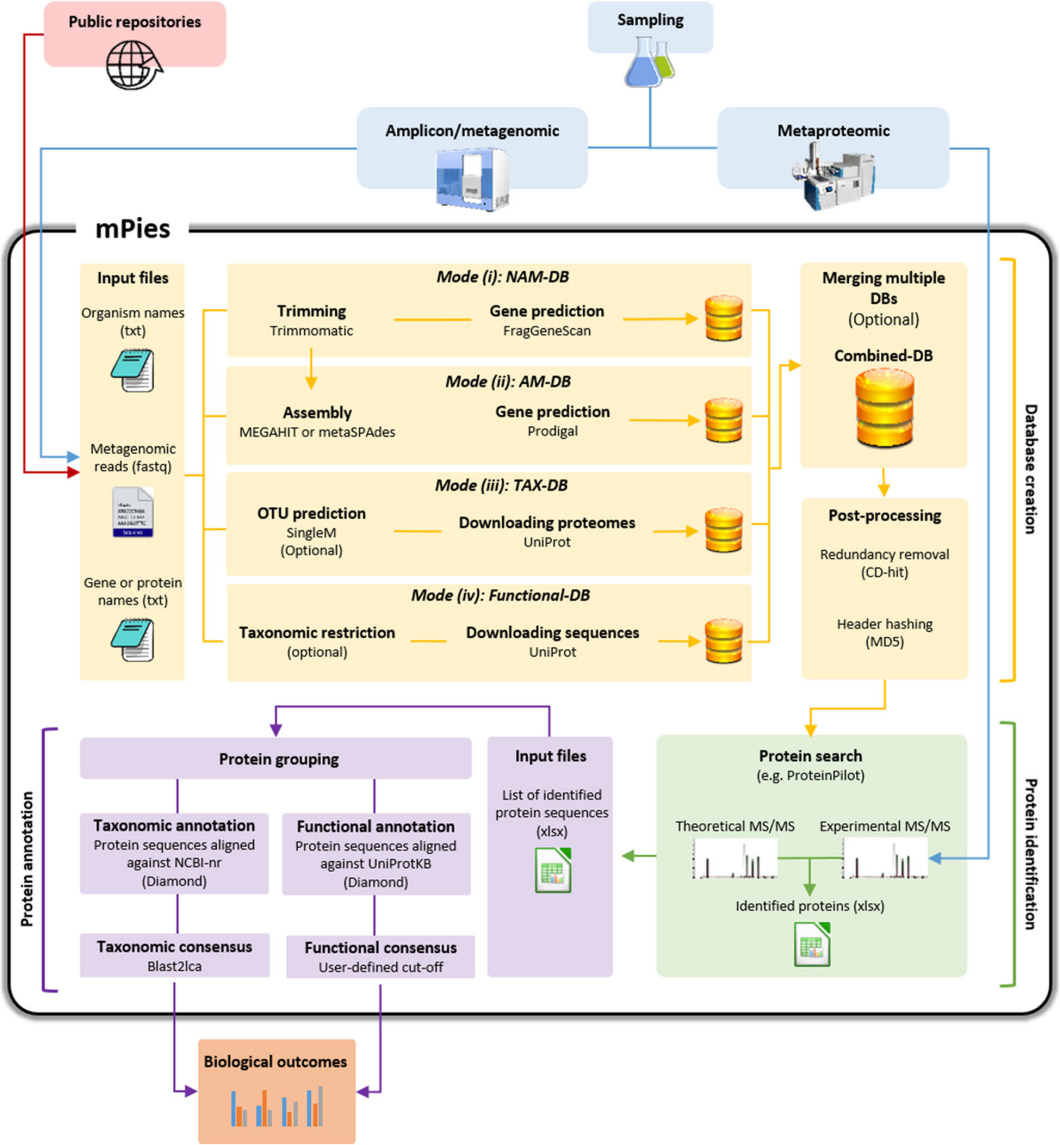
Workflow of mPies

mPies provides multiple options for optimizing metaproteomic analysis within a standardized and automatized workflow (Fig. 1). mPies is written in Python 3.6, uses the workflow management system Snakemake [15] and relies on Bioconda [16] to ensure reproducibility. mPies can run in up to four different modes to create databases (DBs) for protein search using amplicon/metagenomic and/or public repositories data: (i) non-assembled metagenome-derived DB, (ii) assembled metagenome-derived DB, (iii) taxonomy-derived DB, and (iv) functional-derived DB. After protein identification, mPies can automatically compute sequence alignment-based consensus annotation at protein group level. By taking into account multiple alignment hits for reliable taxonomic and functional inference, mPies limits the protein inference issue and allows more relevant biological interpretation of metaproteomes from diverse environments.

#### Mode (i): Non-assembled metagenome-derived DB

In mode (i), mPies trims metagenomic raw reads (fastq files) with Trimmomatic [17], and predicts partial genes with FragGeneScan [18] which are built into the protein DB.

#### Mode (ii): Assembled metagenome-derived DB

In mode (ii), trimmed metagenomic reads are assembled either with MEGAHIT [19] or metaSPAdes [20]. The genes are subsequently called with Prodigal [21]. The utilization of Snakemake allows easy adjustment of the assembly and gene calling parameters.

#### Mode (iii): Taxonomy-derived DB

In mode (iii), mPies extracts the taxonomic information derived from the metagenomic raw data and downloads the corresponding proteomes from UniProt. To do so, mPies uses SingleM [22] to predict OTUs from the metagenomic reads. Subsequently, a non-redundant list of taxon IDs corresponding to the taxonomic diversity of the observed habitat is generated. Finally, mPies retrieves all available proteomes for each taxon ID from UniProt. It is noteworthy that the taxonomy-derived DB can be generated from 16S amplicon data or a user-defined list.

#### Mode (iv): Functional-derived DB

Mode (iv) is a variation of mode (iii) which allows to create DBs that target specific functional processes (e.g. carbon fixation or sulphur cycle) instead of downloading entire proteomes for taxonomic ranks. For that purpose, mPies requires a list of gene or protein names as input and downloads all the corresponding protein sequences from UniProt. Taxonomic restriction can be defined (e.g. *Proteobacteria*-related sequences only) for highly specific DB creation.

#### Post-processing

If more than one mode was selected for protein DB generation, all proteins are merged into one combined protein search DB. Duplicated protein sequences (default: sequence similarity 100%) are removed with CD-HIT [23]. All protein headers are hashed (default: MD5) to obtain uniform headers and to reduce the file size for the final protein search database in order to keep the memory requirements of downstream analysis low.

#### Protein annotation

mPies facilitates taxonomic and functional consensus annotation at protein level. After protein identification, each protein is aligned with Diamond [24] against NCBI-nr [7] for the taxonomic annotation. For the functional prediction, proteins are aligned against UniProt (Swiss-Prot or TrEMBL) [8] and COG [25]. The alignment hits (default: retained aligned sequences = 20, bitscore ≥80) are automatically retrieved for consensus taxonomic and functional annotation, for which the detailed strategies are provided below.

The taxonomic consensus annotation uses the alignment hits against NCBI-nr and applies the LCA algorithm to retrieve a taxonomic annotation for each protein group (protein grouping comprises the assignment of multiple peptides to the same protein and is facilitated by proteomics software) as described by Huson et al. [13]. For the functional consensus, the alignment hits against UniProt and/or COG are used to extract the most frequent functional annotation per protein group within their systematic recommended names. This is the first time that a metaproteomics tool includes this critical step, as previously only the first alignment hit was kept. In order to ensure the most accurate annotation, a minimum of 20 best alignment hits should be kept for consensus annotation. Nevertheless, this parameter is customizable and and this number could be modified.

## Conclusions

The field of metaproteomics has rapidly expanded in recent years and has led to valuable insights in the understanding of microbial community structure and functioning. In order to cope with metaproteomic limitations, new tools development and workflow standardization are of urgent needs. With regards to the diversity of the technical approaches found in the literature which are responsible for methodological inconsistencies and interpretation biases across metaproteomic studies, we developed the open-source program mPies. It proposes a standardized and reproducible workflow that allows customized protein search DB creation and reliable taxonomic and functional protein annotations. mPies facilitates biological interpretation of metaproteomics data and allows unravelling microbial community complexity.

## Reviewer’s comments

### Wilson Wen Bin Goh PhD, School of Biological Sciences, Nanyang Technological University

**Reviewer summary**

Metaproteomics is a growing area. Although its sister discipline, metagenomics is relatively more mature, metaproteomics is expected to be harder due to the indirect means of assaying peptide information based on the MS. There is a lack of tools for performing metaproteomics analysis. And so, I think the author’s pipelines adds a useful resource. The manuscript is well-written, and to the point, I have no points to add regarding grammar and spell proofing.

Authors response: *We thank Dr. Wilson Wen Bin Goh for his overall very positive review.*

**Reviewer recommendations to authors**

The manuscript runs a bit on the short. While I appreciate the conciseness, I think to get more people interested, inclusion of a case study on application, or possible generic user-routes to get people jumping in and tinkering would be great. I particularly like the idea of integrating functional consensus information automatically with a protein group. I think this helps to establish the coherence of a protein group. For example, in the case of OpenMS, some examples of workflows https://www.openms.de/workflows/, help readers understand the usefulness of the pipelines, and how to integrate it with their needs. As Biology Direct is not a bioinformatics journal per se, this addition would help the readership.

Authors response: *We would like to thank the Reviewer for this comment. We agree with the Reviewer’s suggestion and improved the visualization of the overall metaproteomics worfkow using mPies from data generation to biological interpretation (*Fig. 1*). We also provided copy-paste usage examples, with test-data, on the GitHub repository to get people started quickly, thus maximizing the use of mPies by the widest community.*

**Minor issues**

Looking at the protein annotation figure, is the max of 20 a fixed number? Can this be changed? As for most frequent protein name, is it based on SwissProt ID or the gene symbol?

Authors response: *The value for maximum target sequences is adaptable, as are most parameters in the Snakemake workflow. Based on our experience on several (not-yet-published) in-house datasets, 20 is significantly more robust than lower values (tested: 10, 20, 50, 100); higher values do not capture significantly more functions. Depending on the studied environment and available reference data, a higher value for consensus annotations might be useful, although we recommend to never use a value lower than 20 to limit the influence of outliers and false positives.*

The most frequent protein name is not a gene ID but the “recommended” UniProt protein name, which we use for consensus calculation.

We adapted the respective sentences in the revised manuscript.

## Availability and requirements

**Project name:** mPies

**Project homepage:** https://github.com/johanneswerner/mPies/

**Operating system:** Linux

**Programming language:** Python 3.6

**Other requirements:** Snakemake, bioconda

**License:** GNU GPL v3.0

**Any restrictions to use by non-academics:** none.

## Data Availability

Not applicable.
